# Undetectable unintegrated HIV-DNA in persons with HIV: frequency and determinants

**DOI:** 10.1186/s12879-026-13113-z

**Published:** 2026-03-28

**Authors:** Chiara Orlandi, Benedetta Canovari, Francesco Barchiesi, Mauro Magnani, Marco Bruno Luigi Rocchi, Andrea De Maria, Anna Casabianca

**Affiliations:** 1 Department of Biomolecular Sciences, Section of Biochemistry and Biotechnology, University of Urbino Carlo Bo, Fano (PU), Italy; 2 Laboratorio Covid, University of Urbino Carlo Bo, Fano (PU), Italy; 3 Unit of Infection Diseases, San Salvatore Hospital, AST Pesaro Urbino, Pesaro (PU), Italy; 4 Department of Biomedical Sciences and Public Health, Marche Polytechnic University (UNIVPM), Ancona (AN), Italy; 5 Department of Biomolecular Sciences, Campus Scientifico Enrico Mattei, University of Urbino Carlo Bo, Urbino (PU), Italy; 6 Department of Biomolecular Sciences, Service of Biostatistics, University of Urbino Carlo Bo, Urbino (PU), Italy; 7 Department of Health Sciences, University of Genova, Genova (GE), Italy; 8 S. C. Malattie Infettive, Azienda Sociosanitaria del Levante n. 5, Regione Liguria, Sarzana (SP), Italy

**Keywords:** Blood cells, HIV-1 infection, qPCR, Total HIV-DNA, Unintegrated HIV-DNA, Viral reservoir

## Abstract

**Background:**

Despite suppressive antiretroviral therapy (ART), HIV-1 persists in infected cells as integrated proviral DNA and as more labile unintegrated DNA forms (uDNA). The presence of uDNA has been proposed as a surrogate marker of recent infection events occurring below the detection limits of plasma HIV-1 RNA assays. The aim of this study was to evaluate the performance of an assay for the quantification of uDNA in peripheral whole blood of people with HIV (PWH), with the intent of identifying individuals with no evidence of recent infection events, defined as undetectable uDNA, and to characterize associated clinical and immunological factors.

**Methods:**

Total and unintegrated HIV-1 DNA were quantified by real-time PCR in whole blood samples from 87 chronically treated PWH. Samples were derived from a real-world, retrospective cohort and reflected the antiretroviral regimens actually received by patients over time. Clinical, virological, and immunological parameters were analyzed in relation to uDNA detectability.

**Results:**

Overall, uDNA was quantifiable in 11/87 (13%) samples, detectable below the assay quantification limit in 52/87 (60%), and undetectable (target not detected) in 24/87 (28%). Undetectable uDNA was associated with higher CD4⁺ T-cell nadir, higher current CD4⁺ T-cell counts, and lower total HIV-1 DNA levels. In multivariable analysis, CD4⁺ T-cell nadir was the only factor independently associated with uDNA undetectability (*p* = 0.001). Antiretroviral regimen characteristics were not independently associated with uDNA detectability.

**Conclusions:**

A substantial proportion of chronically treated PWH exhibited undetectable uDNA in peripheral blood. The uDNA measurement provides complementary information to plasma HIV-1 RNA and CD4⁺ T-cell counts and may help identify individuals with no evidence of recent infection events and higher immunological fitness. These findings support the potential utility of uDNA quantification as a biomarker of HIV-1 reservoir dynamics in real-world clinical settings.

**Supplementary Information:**

The online version contains supplementary material available at 10.1186/s12879-026-13113-z.

## Introduction

Combined antiretroviral therapy (cART) effectively suppresses the HIV-1 viral load, preserves or improves immune function, and reduces the risk of opportunistic infections and cancers. While cART can reduce the plasma viral load below the detection limits of commercial clinical assays (20–50 copies HIV-1 RNA/mL), it does not eliminate integrated HIV-1 proviral DNA, which contributes to persistent inflammation and viral rebound upon cART discontinuation, within a relatively short time frame in most individuals (2–4 weeks) [1]. Although most of the HIV-1 reservoir consists of nonreplication-competent proviruses, 2–3% are replication-competent [2, 3]. Furthermore, many proviruses contain deletions, and a significant proportion are transcription competent, producing viral proteins that contribute to inflammation in virologically suppressed PWH [4–6].

Quantifying HIV-1 reservoirs, including both integrated (iDNA) and unintegrated HIV-1 DNA (uDNA, i.e., the ensemble of extrachromosomal viral cDNAs, including both linear cDNA and all the closed circular 1-LTR and 2-LTR and other rearranged forms), is crucial for evaluating potential functional cures and targeting transcription-competent defective viruses. In both resting and activated CD4 + T-cells, uDNA is the predominant form of HIV-1 DNA and is detectable in vivo [7–9]. Although these forms of HIV-DNA are labile intermediates, their detection could be indicative of recent *de novo* infection [8, 10]. Thus, uDNA presence, particularly in PWH with a viremia < 50 copies/mL, signals recent infection events and uncontrolled replication below plasma RNA detection limits.

The quantification of uDNA forms has been proposed as biological marker of *de novo* infection and several approaches to measure only the 1-LTR or 2-LTR circles have been developed [11–14], differently, we developed a qPCR-based assays (U-assay) [15], able to simultaneously measure both total HIV-DNA and the totality of the different forms (both linear and circular) of unintegrated HIV-DNA. In previous research we demonstrated that uDNA constitutes a significant proportion of total HIV-DNA: 33% in untreated PWH and 24% in treated PWH with plasma viral load < 50 copies/mL. Compared with the 2-LTR assay (able to detect only the 2-LTR circle forms) the U-assay provides a more accurate measurement of the totality of the uDNA, particularly when the 2-LTR forms are below the quantification limit of the method, being only a minor fraction of the totality of the uDNA [16]. Measuring uDNA offers insights into recent replication events that are not obtainable from other proviral DNA assays. Given the interindividual variability in reservoir size and recent replication episodes in cART-treated PWH, uDNA measurement in real-world cohorts could provide useful information on residual replication and guide therapeutic decisions [17].

Here, to verify the performance of our previously developed U-assay in a real-life context, we measured the uDNA levels in a cohort of 87 PWH under any antiviral treatment with the aim of identifying those with no presence of unintegrated HIV-DNA (undetectable uDNA) in peripheral whole blood and the clinical and immunovirological factors associated with undetectable uDNA.

## Materials and methods

### Participants and sample collection

The selection of 87 anonymous PWH (Table 1), was based on availability of whole blood samples obtained from frozen residual specimens after completion of the analyses requested for clinical purposes for HIV management (HIV-RNA plasma viremia, CD4 + count, etc.). Participants were followed up at the Unit of Infection Diseases of the San Salvatore Hospital (AST Pesaro Urbino), the central reference hospital that managed all PWH residing in the Pesaro Urbino province.

For all samples, a single time point quantification of both total and unintegrated HIV-DNA was performed. Moreover, 18 patients, on the basis of blood sample availability (two consecutive blood samples), were followed for a time dependent analysis. After the cross-sectional analysis of the different forms of HIV-DNA, a retrospective reconstruction of the therapy had been performed. By reviewing records, it has been found that 66 (76%) PWH were receiving only one (*n* = 38) or two (*n* = 28) drugs and the remaining 21 (24%) PWH were receiving ≥ 3 drugs (Tables S1 and S2). Thus, the blood samples/patients were divided into 2 groups on the basis of “unconventional” antiretroviral treatment: those from PWH receiving ≥ 3-drug regimen (Group A) and those from PWH receiving ≤ 2-drug regimen (Group B). PWH from Group A had 7 (IQR: 0.5–16) years of antiretroviral treatment, and those from Group B 17 (IQR: 8–21) years. Blood samples were collected between 2013 (September-December) and 2017 (March-December), particularly those from Group B after 31 (17–47) months of switch from ≥ 3-drugs to a less drug regimen, in accordance with evidence in the literature published in the period in which the samples were collected [18, 19]. It should be noted that this grouping is unrelated to 2-drug or 3-drug regimens referred to by current guidelines and clinical practice [20, 21]. Rather it only reflected the absolute number of drugs on the patient antiretroviral regimens.

A further stratification was based on the type of regimens INSTI and PI. The 11 (13%) PWH under an INSTI-based regimen were 1 (9%) in Group A and 10 (91%) in Group B, the 72 (83%) PWH on PI-based regimen were 11 (15%) in Group A and 61(85%) in Group B (Table S2).

**Table 1:** Characteristics of the PWH included in the study

Characteristics		Overall *n* = 87	Group A (≥ 3 drugs) *n* = 21	Group B (≤ 2 drugs) *n* = 66	Missing	*p* value
Gender	Male n (%)	63 (72%)	16 (76%)	47 (71%)	0 (0%)	0.7831^a^
	Female n (%)	24 (28%)	5 (24%)	19 (29%)		
Age		53 [49–60]	49 [47–57]	54 [51–60]	0 (0%)	0.0816^b^
HIV risk factor					0 (0%)	0.8018^a^
Heterosexual		27 (31%)	7 (33%)	20 (30%)		
MSM		20 (23%)	6 (29%)	14 (21%)		
IVDU		23 (36%)	4 (19%)	19 (29%)		
Unknown		17 (20%)	4 (19%)	13 (20%)		
AIDS diagnosis		27 (31%)	7 (33%)	20 (30%)	0 (0%)	0.7922^a^
Years from diagnosis		17 [7–24]	7 [2–18]	21 [10–24]	0 (0%)	**0.0003** ^b^
CD4 + at nadir (cells/µL)		216 [98–435]	148 [33–300]	240 [115–448]	3 (3.4%)	**0.0468** ^b^
≤ 350		58 (69%)	16 (80%)	42 (66%)		0.2771^a^
≤ 200		38 (45%)	14 (70%)	24 (38%)		**0.0192** ^a^
CD4 + count (cells/µL)		749 [575–1081]	571 [259–816]	784 [597–1118]	2 (2.3%)	**0.0013** ^b^
CD4+ %		36 [29–42]	29 [16–40]	37 [32–43]	5 (5.7%)	**0.0227** ^b^
CD4/CD8 ratio		1.03 [0.77–1.234]	15/21 NA *n* = 6, 0.45 [0.36–0.75]	1.07 [0.83–1.42]	22 (25%)	**0.0024** ^b^
HIV-1 RNA					2 (2.3%)	
< 50 copies/mL of plasma		74 (87%)	21 (100%)	53 (83%)		
> 50 copies/mL of plasma		11 (13%)	0 (0%)	11 (17%)		
HIV-1 RNA at zenith (copies/mL of plasma)		10^5	1.65 × 10^5	10^5	12 (14%)	0.0856^b^
		[10^4-1.8 × 10^5]	[3.7 × 10^4-4.8 × 10^5]	[10^4–10^5]		
Years with suppressed viremia < 50 copies/mL		7 [4–14]	5 [0.5–10]	8 [5–14]	0 (0%)	**0.0173** ^b^
Years on antiretroviral therapy		16 [7–20]	7 [0.5–16]	17 [8–21]	0 (0%)	**< 0.0001** ^b^
Time from the switch (months)				31 [17–47]	0 (0%)	

The results are presented as medians [IQRs] for continuous variables or frequencies (%) for categorical variables. NA, not available

^a^ By Fisher’s exact test or Chi-square test for independence, ^b^ the Mann‒Whitney test, as appropriate. No *p* value was calculated for plasma HIV-1 RNA categories because no participants in Group A had values > 50 copies/mL

### Cellular DNA extraction and unintegrated HIV-DNA isolation

Cellular DNA was isolated from leukocytes of frozen whole blood sample (400 µl) by a DNA extraction kit following the manufacturer’s instructions (QIAGEN QIAamp Blood Mini Kit). The concentrations of the DNA samples were determined by a NanoVue Plus Spectrophotometer (GE Healthcare). The ratios of absorbance at 260/280 nm and at 260/230 nm were used to assess the purity of the DNA, and all the samples had a ratio of > 1.8 and were considered ‘‘pure’’ DNA. Unintegrated HIV-DNA was obtained from cellular DNA via a separation procedure that selectively separated high-molecular-weight cellular DNA (HMW DNA) from low-molecular-weight DNA (LMW DNA). Briefly, the totality of unintegrated HIV-DNA (the ensemble of linear and circular forms of HIV-1) was purified from 5 µg of cellular DNA using the QIAprep Spin Miniprep Kit (Qiagen), adapting this plasmid DNA purification through column chromatography on silica gel separation according to the manufacturer’s instructions and the recommended modifications used for the isolation of low-copy number plasmids. LMW DNA containing uDNA was present in the eluate fraction. To monitor for cross-contamination, one sample of H_2_O in place of DNA and one HIV-1 negative DNA were processed in each separation run. Appropriate experiments to assess the amount, the recovery of the uDNA forms and the extent of DNA degradation have demonstrated the feasibility of the procedure [15, 22].

### Measurement of total and unintegrated HIV-DNA

Total and unintegrated HIV-DNA were simultaneously analysed via a SYBR Green qPCR-based method in a single run using a single set of specific primers selected in the 5’ LTR-Gag region of the HIV-1 genome, including the highly conserved primer-binding site (PBS), resulting in an amplicon with a size of 161 bp (PBS fragment); this method can detect all HIV-1 subtypes in the M group [15–17]. PCRs were carried out in a 7500 real-time PCR system (Applied Biosystems, Thermo Fisher Scientific, Inc.) using TB Green Premix Ex Taq (TaKaRa Bio, Inc.). Each sample (cellular DNA or eluate fraction) was analysed in six replicates, consisting of three wells containing 0.5 µg of DNA or the equivalent quantity of the elution fraction and three wells containing 1.0 µg of DNA. For samples with an HIV-DNA datum measured near or detected below the quantification limit (QL) of PCR (QL: 2 copies/PCR), two additional 1 µg replicates were tested for a total of 6.5 µg of DNA (~ 10^6 leukocytes, WBC, to ensure the detection of the target even at low copy numbers). For negative amplification, a PCR spike test was performed by adding 2 or 10 copies of the pPBS plasmid standard (161 bp PBS fragment cloned in the pGEM-T vector) to the PCR mixture. The positive amplification excluded the presence of inhibitors and confirmed that no HIV-DNA target was present in the sample. The HIV-DNA copy number was quantified by interpolating the experimentally determined threshold cycle (Ct) based on the standard curve generated by half-log serial pPBS plasmid dilutions from 10^5 to 2 copies and by adding the copy number from the 0.5 and 1.0 µg replicates and finally expressed as copies/10^4 CD4 + T-cells. This approach assumes that 1 µg of genomic DNA corresponds to 142,857 cells [23] and that HIV-DNA is present principally in CD4 + T lymphocytes [24]. Hence, the following formula was used: copies/10^4 CD4+ = [(copies/µg DNA)/(CD4 + T-cell count (/µL)/WBC count (/µL) × 142,857 WBC)] × 10^4. For samples with HIV-DNA less than 2 copies, an imputed value corresponding to ½QL (1 copy) was used for subsequent normalization and statistical analyses. When no amplification signal was revealed by the real time PCR instrument, we assumed 0 copies of the 161 bp of HIV-1 genome fragment and the sample was defined as undetectable (TND, target not detected).

The amount of integrated HIV-DNA can be obtained by subtracting the amount of uDNA from the amount of total HIV-DNA [22].

### Quantification of the plasma HIV‑1 RNA concentration and CD4 + T-cell count

Plasma HIV-1 RNA levels were quantified using commercial kit according to the manufacturer’s instructions (artus HI Virus-1 QS-RGQ Kit Qiagen, or Nuclisens EasyQ HIV-1 2.0 bioMérieux SA). CD4 + T lymphocyte counts were determined using flow cytometry analysis.

### Statistical analysis

The data are presented as medians and interquartile ranges (IQRs, 25th to 75th percentiles) for continuous variables or frequencies (%) for categorical variables. Fisher’s exact test, the Mann‒Whitney test (for two-group unpaired data), and the Wilcoxon signed rank test (for two-group paired data) were used for comparisons of values between groups. Pearson correlation test was used to assess correlations between variables and total HIV-DNA and uDNA.

For multivariate analysis, logistic regression was used to assess factors associated with undetectable (TND, target not detected) uDNA; adjusted odds ratios (aORs) with their respective 95% confidence intervals (95% CI) were reported. The significance level was set at 0.05 (two-sided) for all tests. All the analyses were performed using SPSS 23.0 software (SPSS Inc., Chicago, IL, USA). GraphPad Prism (version 8.4.2, GraphPad Software, San Diego, CA, USA) was used to draw the plots.

## Results

### Characteristics of the PWH

The study included 21 (24%) PWH receiving ≥ 3 drugs (Group A) and 66 (76%) PWH receiving ≤ 2-drugs (Group B). The rationale for the type of antiretroviral treatment and the quantity of medications taken (one, two, three, or more) was based on a strict clinical criterion for each PWH, justified by the patient’s individual needs (i.e., intolerances, comorbidities), and not according to standard therapeutic protocols [20, 21].

PWH receiving ≥ 3- and ≤ 2-drug regimens were characterized by long-lasting HIV-1 infection with a median of 17 years from diagnosis (IQR: 7–24) and a median antiretroviral treatment duration of 16 years (IQR: 7–20). Clinical and virological parameters are summarized in Table 1. The median age of the overall population was 53 years [49–60], and 72% of the subjects were men. Notably, CD4 + T-cell counts, nadir CD4+, CD4 + percentages and CD4/CD8 ratios were significantly greater in PWH from Group B (*p* ≤ 0.0468).

### Analysis of the HIV-1 reservoir size

**Fig. 1:**
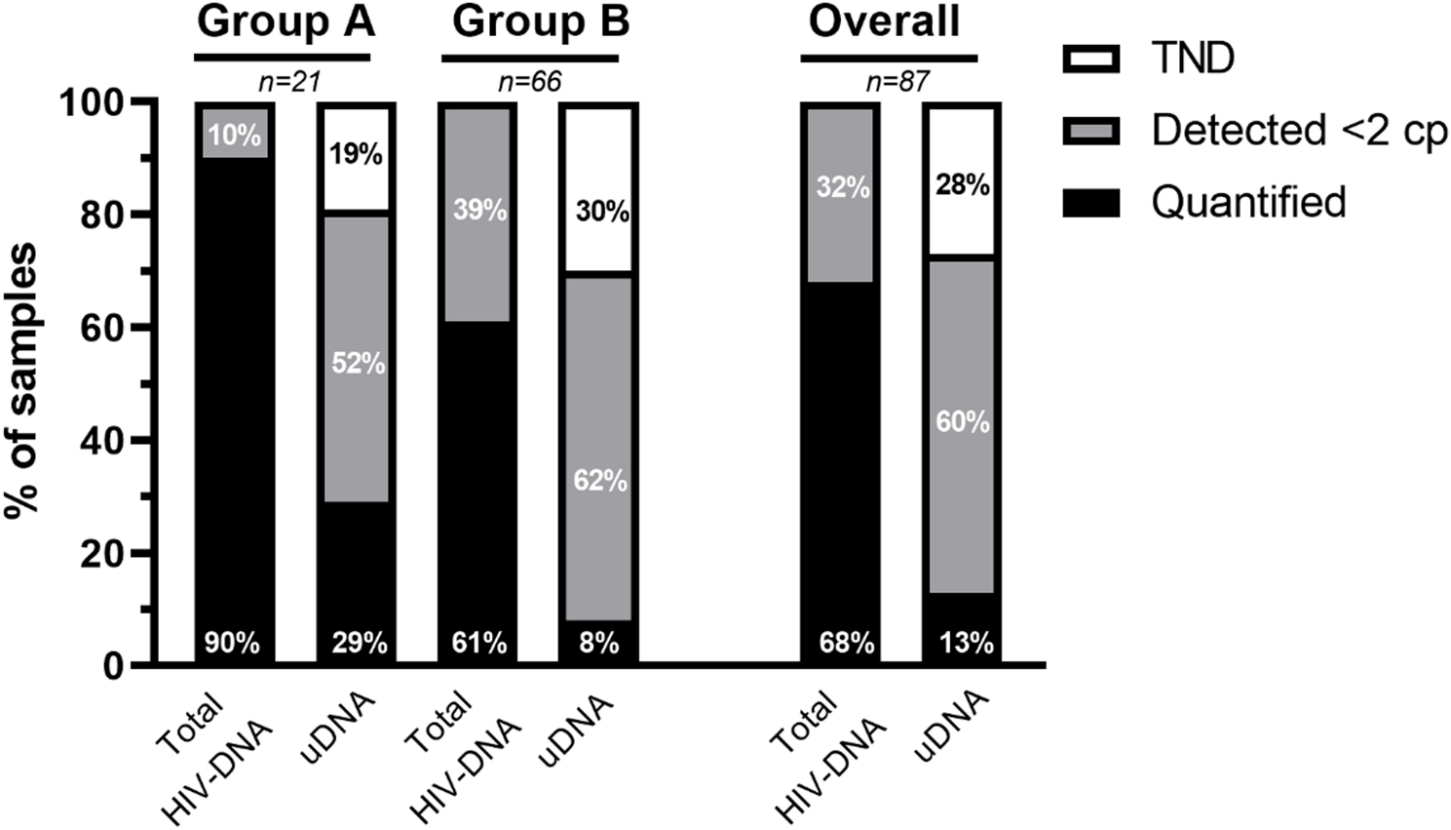
Proportion of samples from PWH exhibiting quantifiable, detectable (below the assay’s quantification limit of 2 copies), or undetectable (target not detected [TND]) levels of total and unintegrated HIV-DNA. Group A: PWH receiving ≥ 3 drug regimen, Group B: PWH receiving ≤ 2-drug regimen

The distribution of HIV-1 reservoir size, on the basis of the presence of different HIV-1 DNA forms, was determined for all PWH and is presented in Fig. 1, which provides an estimate of their relative proportions. Overall, total HIV-DNA was detectable in all the samples. However, quantification was possible in 68% of the samples (59/87), while 32% (28/87) exhibited levels below the assay quantification limit (QL) of 2 copies. To assess the relative abundance of total HIV-DNA and uDNA, the analysis was further stratified by the number of drugs (≥ 3 drugs vs. ≤2 drugs), showing that 10% of Group A samples (2/21) and 39% of Group B samples (26/66) presented total HIV-DNA levels below the QL.

Overall, with respect to uDNA, a marker of recent HIV-1 reservoir expansion, only 13% of the samples (11/87) had quantifiable uDNA, 60% (52/87) presented levels below the QL, and the remaining 28% (24/87) had no quantifiable uDNA (TND). The distribution of quantifiable uDNA was 29% in Group A samples (6/21) and 8% in Group B samples (5/66). Levels below the QL were found in 52% of the Group A samples (11/21) and in 62% of the Group B samples (41/66). The remaining 19% (4/21) and 30% (20/66) of the samples (Group A and Group B, respectively) had undetectable uDNA.

**Fig. 2:**
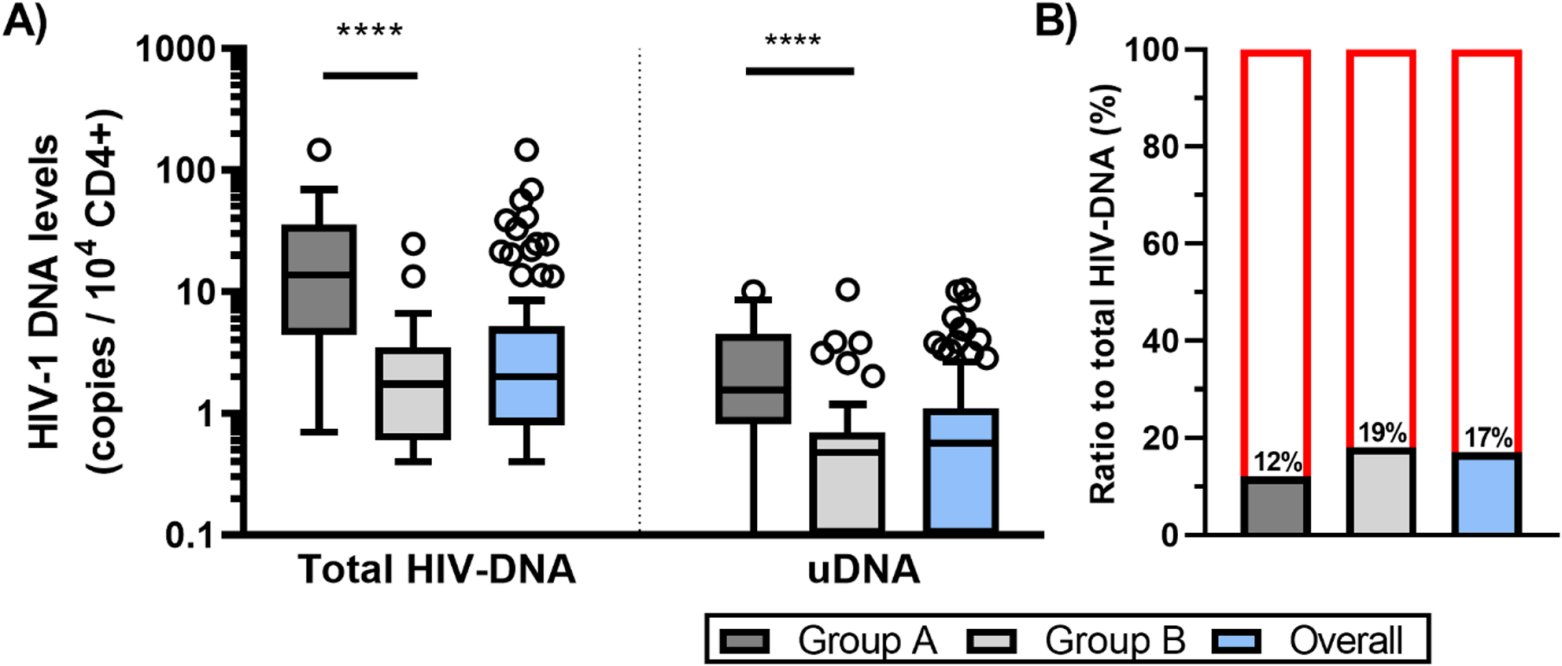
**(A)** Levels of total and unintegrated HIV-DNA in two groups of PWH. Group A: PWH receiving ≥ 3 drug regimen, Group B: PWH receiving ≤ 2-drug regimen). **(B)** Percentages of uDNA among total HIV-DNA in each group and overall. Subtracting uDNA from total HIV-DNA, the remaining fraction consists of integrated HIV-DNA (red line). ****p < 0.0001, Mann‒Whitney test

Compared with samples from PWH receiving ≥ 3 drugs (Group A), samples from PWH receiving ≤ 2 drugs (Group B) presented significantly lower levels of HIV-1 DNA. Specifically, total HIV-DNA levels were 8-fold lower (median [IQR] 14 [4.4–36] vs. 1.8 [0.6–3.5] copies/10^4 CD4+, *p* ≤ 0.0001), and uDNA levels were 3-fold lower (median [IQR] 1.6 [0.8–4.5] vs. 0.5 [0–0.7] copies/10^4 CD4+, *p* ≤ 0.0001) in Group B (Fig. 2A). While the uDNA-to-total HIV-DNA ratio was slightly greater in Group B, this difference was not statistically significant (median [IQR] 12% [4.5–28] vs. 19% [0–50], *p* = 0.3612) (Fig. 2B).

The comparison analysis of samples from PWH under INSTI-based vs. no-INSTI, and PI-based vs. no-PI regimens revealed no significant difference in the content of total HIV-DNA (*p* = 0.6796 and *p* = 0.2899), uDNA (*p* = 0.8762 and *p* = 0.4871), or the ratio uDNA-to-total HIV-DNA (*p* = 0.9313 and *p* = 0.1515), (Fig S1).

### Analysis of the HIV-1 reservoir size during follow-up

**Fig. 3:**
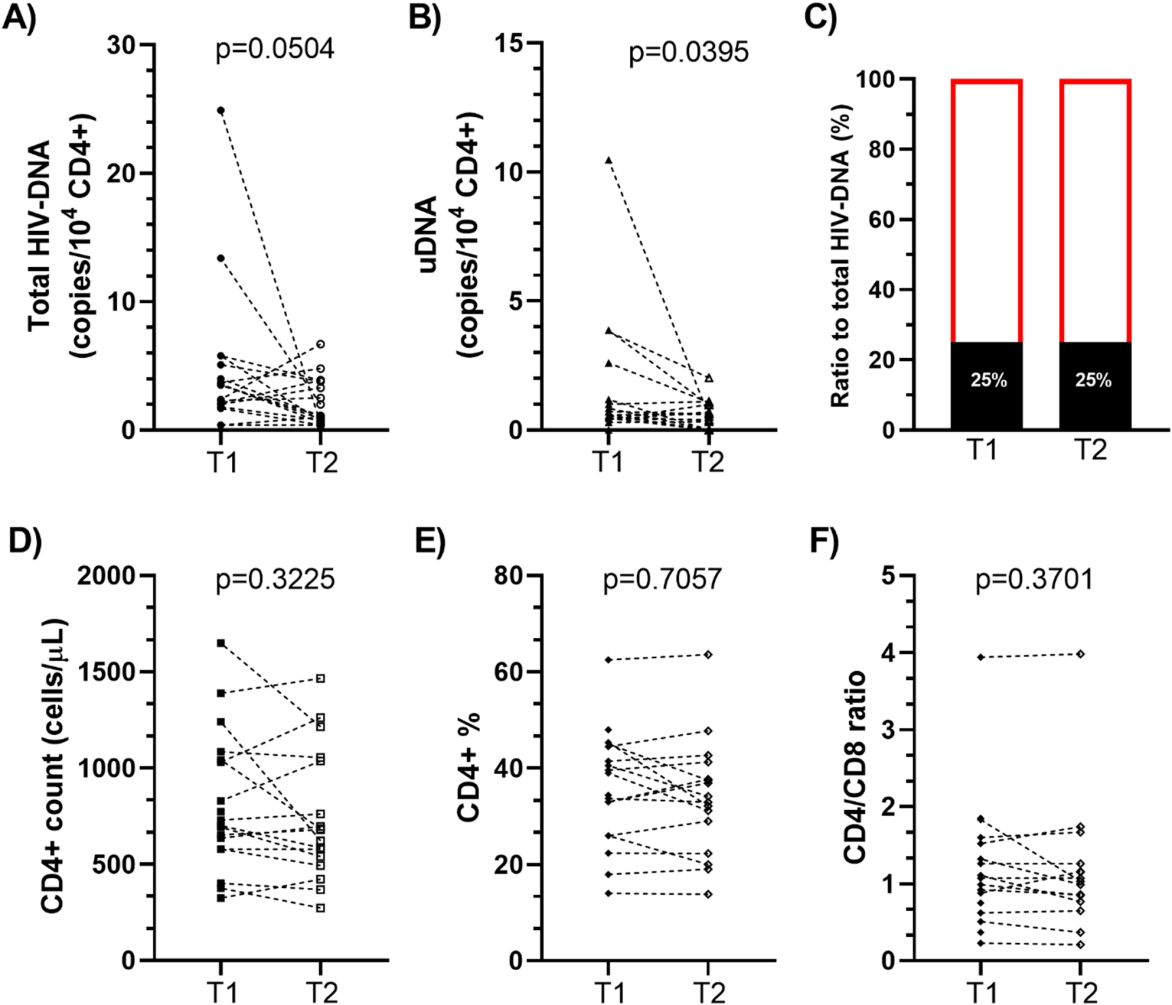
Levels of total **(A)** and unintegrated **(B)** HIV-DNA in a subset of 18 PWH receiving ≤ 2-drug regimen (Group B) for whom two consecutive blood samples were available. **(C)** Percentages of uDNA among total HIV-DNA at each time point. When uDNA is subtracted from total HIV-DNA, the remaining fraction consists of integrated HIV-DNA (red line). **(D)** CD4 + T-cell count, **(E)** CD4 + percentage and **(F)** CD4/CD8 ratio at each time point. T1 and T2 refer to a median visit time of 6 [5–7] months. Wilcoxon matched-pairs signed rank test

To evaluate the potential applicability of the U-assay in the monitoring of uDNA level variations, a time-dependent analysis was conducted on a subset of PWH on the basis of blood sample availability. For 18 PWH receiving ≤ 2-drug regimen, two consecutive samples were analysed (T1 and T2), at a median intervisit interval of 6 [5–7] months. The dynamics of the HIV-1 reservoir and immune reconstitution parameters are presented in Fig. 3, Tables S3, and S4. This analysis revealed a significant reduction in uDNA (*p* = 0.0395), while no statistically significant change was observed for total HIV-DNA (*p* = 0.0504); the uDNA-to-total HIV-DNA ratio remained stable. No significant changes in the CD4 + T-cell count (*p* = 0.3225), CD4 + T-cell percentage (*p* = 0.7057), or CD4/CD8 ratio (*p* = 0.3701) were observed between T1 and T2. With respect to plasma HIV-1 RNA levels, 13 PWH maintained viral suppression (< 50 copies/mL) throughout the observation period, two experienced an increase from < 50 to 92 and 644 copies/mL, two remained viremic (with levels changing from 50738 to 429 and from 377 to 685 copies/mL), and one with 60 copies/mL achieved viral suppression. During the follow-up, a trend towards a reduction in total HIV-DNA (*p* = 0.0156) and uDNA (*p* = 0.0781) was observed in the subgroup of 13 virally suppressed PWH but not in the remaining 5 unsuppressed PWH (*p* > 0.9999 for total HIV-1 DNA and *p* = 0.25 for uDNA). Both subgroups maintained stable immunological parameters during follow-up (*p* > 0.25). Viremic PWH had total HIV-1 DNA levels comparable to those of suppressed PWH at T1 (*p* = 0.5160) but approximately 4-fold higher at T2 (*p* = 0.0626). The levels of uDNA were nearly doubled in viremic PWH at both T1 and T2, although the difference was not statistically significant (*p* = 0.6857 and *p* = 0.5140, respectively). The CD4 + T-cell count, CD4 + T-cell percentage and CD4/CD8 ratio were similar in both groups at both time points (Table S4).

### Correlation of total HIV-DNA and uDNA with demographic and viro-immunological parameters

Unintegrated HIV-1 DNA (uDNA) levels exhibited a strong positive correlation with total HIV-DNA (*r* = 0.67, *p* < 0.0001), accounting for 17% [0–36] of total HIV-DNA. This association persisted in both groups: Group A (*r* = 0.66, *p* = 0.0012), where uDNA represented 12% [4.5–28] of total HIV-DNA, and Group B (*r* = 0.86, *p* < 0.0001), where uDNA constituted 19% [0–50] of total HIV-DNA (Fig. 2B).

As expected, an inverse relationship was observed between the HIV-1 reservoir size and both years of antiretroviral therapy (ART) (*r*=-0.359 and *r*=-0.343, *p* = 0.001 for total HIV-DNA and uDNA, respectively) and years since HIV-1 diagnosis (*r*=-0.343, *p* = 0.001 and *r*=-0.314, *p* = 0.003 for total HIV-1 DNA and uDNA, respectively). A positive correlation was detected between the zenith HIV-1 RNA level and both total HIV-DNA (*r* = 0.413, *p* < 0.0001) and uDNA (*r* = 0.274, *p* = 0.017) levels. Conversely, a negative correlation was observed between years of stable viral suppression (< 50 copies/mL) and both total HIV-DNA (*r*=-0.310, *p* = 0.003) and uDNA (*r*=-0.326, *p* = 0.002) levels. In addition, PWH age was not associated with total HIV-DNA (*r* = 0.093, *p* = 0.392) and weakly positively correlated with uDNA levels (*r* = 0.268, r^2^= 0.072, *p* = 0.012). Table 2 presents the correlations between immunological parameters and the HIV-1 reservoir. As expected, in the overall PWH population, both total HIV-DNA and uDNA were negatively correlated with CD4 + T-cell counts. These associations did not reach statistical significance in the individual groups, except for uDNA in Group A (*p* = 0.0031). In the overall PWH population but not in individual groups, nadir CD4 + cell counts tended to be negatively associated with both total HIV-DNA (*p* = 0.0556) and uDNA (*p* = 0.0696). CD4 + percentages were negatively correlated with total HIV-DNA only in the overall PWH population, whereas uDNA exhibited a stronger negative correlation in both individual groups, particularly in Group A. Although CD4/CD8 ratio data were limited in Group A (available for only 6 of 21 PWH, 29%), the association between this parameter and the HIV-1 reservoir was assessed, revealing that the CD4/CD8 ratio was inversely associated with uDNA (*p* = 0.0093). Supplemental Table S5 shows all the relationships between the parameters that were included in the analysis.

**Table 2:** Correlations between reservoir and immune reconstitution parameters

		CD4 + T-cell count		CD4 + at nadir		CD4+ %		CD4/CD8 ratio	
		Pearson r	*p* value	Pearson r	*p* value	Pearson r	*p* value	Pearson r	*p* value
Total HIV-DNA	Overall	-0.352	**0.0010**	-0.210	0.0556	-0.371	**0.0006**	-0.227	0.0687
	Group A	-0.387	0.0827	-0.239	0.3097	-0.384	0.0948	-0.480	0.3348
	Group B	-0.237	0.0593	-0.132	0.2981	-0.202	0.1153	-0.136	0.3055
uDNA	Overall	-0.396	**0.0002**	-0.199	0.0696	-0.495	**< 0.0001**	-0.320	**0.0093**
	Group A	-0.614	**0.0031**	-0.345	0.1359	-0.674	**0.0011**	-0.265	0.6118
	Group B	-0.147	0.2478	-0.014	0.9132	-0.225	0.0790	-0.281	**0.0311**

### Predictors of uDNA undetectability

The 87 PWH were further divided on the basis of reservoir expansion as determined via unintegrated HIV-1 DNA measurement. The characteristics of PWH stratified by uDNA detectability (quantifiable and detectable uDNA) vs. undetectability are summarized in Table 3. PWH with quantifiable and detectable uDNA were more frequently male (49/63, 78%, *p* = 0.1058) and older (*p* = 0.0135), presented lower nadir CD4 + cell counts (with over half having ≤ 200 CD4 + cells at nadir, *p* = 0.0013), and had higher total HIV-1 DNA levels (*p* < 0.0001) than PWH with undetectable uDNA. Multivariate logistic regression analysis revealed that the nadir CD4 + cell count was the sole protective factor against having quantifiable uDNA levels after adjusting for other variables (*p* = 0.008, Table 4, Model 1). Furthermore, after excluding variables with *p* > 0.5, the CD4 + T-cell count (*p* = 0.027) and years from diagnosis (*p* = 0.037) were also associated with undetectable uDNA (Table 5, Model 2). Sex, age, therapy duration or type, zenith HIV-1 RNA, duration of controlled viral load, and total HIV-1 DNA were not significantly associated. When only years from diagnosis, the CD4 + T-cell count, and nadir CD4 + cell count were included in the third model, the nadir CD4 + cell count remained the only significant predictor (*p* = 0.001, Table 6, Model 3), indicating its primary relevance in association with undetectable uDNA.

**Table 3:** Characteristics of PWH according to unintegrated HIV-DNA undetectability groups

Characteristics		Undetectable uDNA levels (*n* = 24)	Quantifiable and detectable uDNA levels (*n* = 63)	*p* value
Gender	Male n (%)	14 (58%)	49 (78%)	**0.0020** ^a^
	Female n (%)	10 (42%)	14 (22%)	
Age		50 [47–56]	55 [52–62]	**0.0135** ^b^
HIV risk factor				0.7919^a^
Heterosexual		9 (38%)	18 (29%)	
MSM		6 (25%)	14 (22%)	
IVDU		5 (21%)	18 (29%)	
Unknown		4 (17%)	13 (21%)	
AIDS diagnosis		4 (17%)	23 (37%)	0.1184^a^
Years from diagnosis		14 [7–22]	18 [7–25]	0.6517^b^
CD4 + at nadir (cells/µL)		315 [217–414]	176 [63–440], 3 NA	0.0559^b^
≤ 350		16 (67%)	42 (70%)	0.7976^a^
≤ 200		4 (17%)	34 (57%)	**0.0013** ^a^
CD4 + count (cells/µL)		777 [547–1120], 1 NA	736 [578–1079], 1 NA	0.6531^b^
CD4+ %		38 [33–42], 1 NA	33 [26–42], 4 NA	0.1117^b^
CD4/CD8 ratio		1.0 [0.8–1.6], 4 NA	1.0 [0.8–1.2], 18 NA	0.3071^b^
HIV-1 RNA				
< 50 copies/mL of plasma		21 (91%)	53 (85%)	0.7193^a^
> 50 copies/mL of plasma		2 (9%)	9 (15%)	
N.A.		1	1	
HIV-1 RNA at zenith (copies/mL of plasma)		10^5 [10^4–10^5], 2 NA	10^5 [10^4-2.76 × 10^5], 10 NA	0.1902^b^
Years with suppressed viremia < 50 copies/mL		8 [6–15]	7 [2–12]	0.0802^b^
Years on antiretroviral therapy		14 [7–20]	17 [7–21]	0.5686^b^
Therapy				0.4069^a^
≤ 2-drugs		20 (83%)	46 (73%)	
≥ 3-drugs		4 (17%)	17 (27%)	
Total HIV-DNA (copies/10^4 CD4+)		0.7 [0.5–1.6]	3.2 [1.6-7]	< 0.0001^b^
uDNA (copies/10^4 CD4+)		0 [0–0]	0.7 [0.5-2]	< 0.0001^b^
Ratio uDNA to total HIV-DNA (%)		0 [0–0]	25 [14–67]	< 0.0001^b^

The results are presented as medians [IQRs] for continuous variables or frequencies (%) for categorical variables. NA, not available

^a^ By Fisher’s exact test or Chi-square test for independence, ^b^ the Mann‒Whitney test, as appropriate

**Table 4:** Logistic regression analyses of the odds of uDNA quantifiability in chronically treated PWH (Model 1)

Variables	Regression coefficient (log-odds)	S.E.	Wald test	df	*p* value	aOR	95% CI for aOR	
							Lower	Upper
Years from diagnosis	− 0.088	0.122	0.520	1	0.471	0.916	0.720	1.164
Age	− 0.070	0.047	2.278	1	0.131	0.932	0.851	1.021
Years on ART	− 0.007	0.176	0.001	1	0.970	0.993	0.703	1.404
Years on stable viremia < 50 copies/mL	0.088	0.110	0.635	1	0.426	1.092	0.880	1.354
Total HIV-DNA/10^4 CD4+	− 0.156	0.130	1.452	1	0.228	0.855	0.663	1.103
Therapy [0 = ≥ 3-drugs, 1 = -≤2-drugs]	1.778	1.527	1.355	1	0.244	5.915	0.296	118.021
Gender [0 = Female, 1 = Male]	− 0.756	0.762	0.984	1	0.321	0.470	0.106	2.091
Therapy [0 = without PI, 1 = PI-based]	-1.811	1.311	1.908	1	0.167	0.163	0.013	2.135
Compliance [0 = Poor, 1 = Good/adequate]	0.917	1.539	0.355	1	0.551	2.503	0.123	51.123
HIV-1 RNA at zenith (copies/mL)	0.000	0.000	0.104	1	0.747	1.000	1.000	1.000
CD4 + T-cell count (cells/µL)	− 0.003	0.001	3.146	1	0.076	0.998	0.995	1.000
CD4+ %	− 0.016	0.053	0.088	1	0.767	0.984	0.888	1.092
CD4 + at nadir (cells/µL) [0 = < 200, 1 = > 200]	3.142	1.176	7.144	1	**0.008**	23.147	2.312	231.797
Constant	4.174	3.576	1.362	1	0.243	64.984		

S.E., standard error; Wald test to determine statistical significance for each independent variable; df, degrees of freedom; aOR, adjusted odds ratio; CI, confidence interval. The Nagelkerke R Square value is 0.512

**Table 5:** Logistic regression analyses of the odds of uDNA quantifiability in chronically treated PWH (Model 2)

Variables	Regression coefficient (log-odds)	S.E.	Wald test	df	*p* value	aOR	95% CI for aOR	
							Lower	Upper
Years from diagnosis	− 0.106	0.051	4.338	1	**0.037**	0.900	0.815	0.994
Age	− 0.069	0.044	2.426	1	0.119	0.933	0.855	1.018
Years on stable viremia < 50 copies/mL	0.108	0.068	2.553	1	0.110	1.114	0.976	1.273
Total HIV-DNA/10^4 CD4+	− 0.166	0.127	1.719	1	0.190	0.847	0.661	1.086
Therapy [0 = ≥ 3-drugs, 1 = -≤2-drugs]	1.869	1.484	1.586	1	0.208	6.484	0.354	118.935
Gender [0 = Female, 1 = Male]	− 0.816	0.717	1.293	1	0.255	0.442	0.108	1.804
Therapy [0 = without PI, 1 = PI-based]	-1.968	1.204	2.670	1	0.102	0.140	0.013	1.481
CD4 + T-cell count (cells/µL)	− 0.003	0.001	4.912	1	**0.027**	0.997	0.995	1.000
CD4 + at nadir (cells/µL) [0 = < 200, 1 = > 200]	2.926	1.029	8.090	1	**0.004**	18.644	2.483	139.971
Constant	4.824	2.541	3.605	1	0.058	124.452		

S.E., standard error; Wald test to determine statistical significance for each independent variable; df, degrees of freedom; aOR, adjusted odds ratio; CI, confidence interval. The Nagelkerke R Square value is 0.505

**Table 6:** Logistic regression analyses of the odds of uDNA quantifiability in chronically treated PWH (Model 3)

Variables	Regression coefficient (log-odds)	S.E.	Wald test	df	*p* value	aOR	95% CI for aOR	
							Lower	Upper
Years from diagnosis	− 0.034	0.029	1.369	1	0.242	0.966	0.912	1.023
CD4 + T-cell count (cells/µL)	− 0.001	0.001	0.943	1	0.331	0.999	0.998	1.001
CD4 + at nadir (cells/µL) [0 = < 200, 1 = > 200]	2.527	0.790	10.224	1	**0.001**	12.510	2.659	58.866
Constant	-1.530	0.803	3.629	1	0.057	0.217		

S.E., standard error; Wald test to determine statistical significance for each independent variable; df, degrees of freedom; adjusted odds ratio; CI, confidence interval. The Nagelkerke R Square value is 0.230

## Discussion

After HIV-1 enters the cell, viral RNA is reverse transcribed into double-stranded DNA, a process that is essential for viral replication. This viral DNA then integrates into the host cell genome and persists as the major component of the HIV-1 reservoir. In parallel, unintegrated viral DNA can also be detected as linear or circular forms, which are generally considered labile byproducts of reverse transcription and unable to sustain productive infection [25–28]. Nevertheless, accumulating evidence indicates that unintegrated HIV-1 DNA may retain limited transcriptional activity and contribute to viral replication and persistence, supporting its biological relevance as a marker of recent infection events [29–31]. In this respect, the role of unintegrated HIV-1 DNA in viral persistence is reinforced by Gurgo et al. showing that the 1-LTR episomal form replicates in the absence of an origin of replication and viral integration [32] and by reports of a mechanism to evade type 1 IFN-mediated antiviral activity involving uDNA via its histone binding domain [33].

Accurate measurement of the HIV-1 reservoir is critical for evaluating therapeutic strategies and understanding viral persistence. Although multiple assays have been developed to interrogate different components of the reservoir (e.g. PCR-based as the intact proviral DNA assay, IPDA [34–37], flow cytometry and microscopy-based methods [38–40], quantitative viral outgrowth assays and modifications [41–44]), no single method fully captures its complexity or dynamic behavior. Furthermore, advanced technologies such as next-generation sequencing may not be readily applicable in routine clinical practice, particularly when the primary aim is quantitative rather than structural characterization [3, 45, 46]. In this context, uDNA measurement may provide complementary information on recent infection events that is not obtainable from plasma HIV-1 RNA or integrated HIV-1 DNA measurements alone.

The quantification of unintegrated HIV-1 DNA forms has therefore been proposed as a surrogate marker of ongoing viral replication [8, 10, 47], and several approaches targeting 1-LTR or 2-LTR circles [8, 11–14] have demonstrated the persistence of episomal HIV-DNA in both untreated and treated individuals [48]. In previous work [15, 16], we applied the uDNA assay to a range of clinical conditions, demonstrating that uDNA is detectable across diverse virological and immunological settings including 100% of the samples from Off-ART, 90% and 76% from viremic and aviremic ART-treated PWH and 45% from HICs (HIV-infected controllers). These findings supported the applicability of this assay for investigating recent infection events in vivo.

The purpose of the present study was to perform a qualitative and quantitative analysis of the HIV-1 reservoir in whole blood samples from chronically treated persons with HIV, focusing on both total HIV-DNA and its unintegrated fraction. The primary aim was to evaluate the performance of the uDNA assay in a real-world context and to identify individuals with no evidence of recent infection events, defined as undetectable uDNA, as well as the clinical and immunological factors associated with this condition.

Accordingly, we found that a substantial proportion of the study population exhibited quantifiable or detectable uDNA, specifically 81% of persons receiving ≥ 3 drugs and 70% of those receiving ≤ 2 drugs. In this cohort, persons receiving ≤ 2 drugs were observed to have lower uDNA and total HIV-DNA levels compared with those receiving ≥ 3 drugs. These findings should be interpreted cautiously, as the study was not designed to compare antiretroviral regimens and reflects a heterogeneous, real-world population. Importantly, these observations do not imply comparative efficacy between treatment strategies.

One possible explanation for these findings may relate to differences in host immune control rather than treatment characteristics per se, as suggested by the strong association with CD4⁺ T-cell counts and CD4⁺ T-cell nadir. Given the retrospective nature of the cohort and its constitution over time, an enrichment of individuals with intrinsically favorable immunological profiles, such as historical elite controllers or long-term non-progressors, who have been shown to better control virus replication independent of antiviral treatment, may have occurred among those receiving ≤ 2 drugs. These hypotheses remain speculative and cannot be formally tested within the design of the present study.

Antiretroviral drug classes may differentially influence uDNA by affecting viral integration, persistence, or host-mediated silencing mechanisms. For example, integrase inhibitors prevent viral DNA integration and may transiently increase unintegrated DNA forms [47, 49, 50], whereas other agents including histone deacetylase (HDAC) inhibitors could increase gene expression and replication of unintegrated HIV-1 DNA [30]. In the present cohort, however, comparisons between INSTI-based versus non-INSTI regimens and PI-based versus non-PI regimens did not reveal significant differences in total HIV-DNA, uDNA, or their ratio, suggesting that drug class did not independently affect uDNA levels in this setting.

A time-dependent analysis was conducted in a subset of 18 persons receiving ≤ 2-drug regimens for whom two consecutive blood samples were available. This exploratory analysis showed largely stable or modestly changing reservoir measures over a limited follow-up period, without significant changes in immunological parameters. Given the small sample size and exploratory nature of this analysis, these longitudinal observations should be considered hypothesis-generating rather than confirmatory.

Correlation analyses revealed that uDNA levels were strongly associated with total HIV-DNA and inversely associated with immunological markers, including CD4⁺ T-cell nadir and current CD4⁺ T-cell counts, frequency and CD4/CD8 ratio. The uDNA also showed significant associations with several clinical and virological variables, but weakly with PWH age.

In multivariable analysis, CD4⁺ T-cell nadir emerged as the sole independent factor associated with uDNA undetectability, underscoring its central role in long-term immune control of HIV-1 infection. Other variables, including treatment type, treatment duration, sex, age, and plasma HIV-1 RNA levels, were not independently associated with uDNA detectability.

Several limitations of this study should be acknowledged. First, the relatively small number of samples, selected based on availability, may limit statistical power, particularly for subgroup analyses, and the generalizability of our findings. Second, the retrospective design and heterogeneity of treatment histories limit causal inference, so results that did not reach statistical significance were interpreted descriptively without inferential emphasis. In addition, HIV-DNA measurements were restricted to peripheral blood and may not fully reflect reservoir dynamics in other anatomical compartments [51, 52]. Nevertheless, whole blood represents the most accessible tissue for routine clinical monitoring and has been shown to reasonably reflect systemic HIV-1 reservoir burden [53].

In conclusion, this study reinforces the relevance of unintegrated HIV-DNA as a surrogate biomarker of recent infection events in chronically treated persons with HIV. The uDNA measurement may complement established virological and immunological markers by providing additional insight into reservoir dynamics and immune control. Prospective studies and structured longitudinal analyses are warranted to further clarify the potential clinical utility of uDNA quantification.

## Supplementary Information

Below is the link to the electronic supplementary material.

Supplementary Material 1

## Data Availability

All the data generated or analysed during this study are included in this published article and its additional files.
