# Supplementary material for: Undetectable unintegrated HIV-DNA in persons with HIV: frequency and determinants
Source: BMC Infect Dis. 2026 Mar 28;26:914. doi: 10.1186/s12879-026-13113-z (PMC13151317; doi:10.1186/s12879-026-13113-z)
Supplement: Supplementary file 1 — Supplementary Material 1 [file 12879_2026_13113_MOESM1_ESM.docx]

**-Supplementary Information-**

**Undetectable unintegrated HIV****-DNA in** **persons with HIV: frequency and determinants**

Chiara Orlandi^1,2^, Benedetta Canovari^3^, Francesco Barchiesi^3,4^, Mauro Magnani^5^, Marco Bruno Luigi Rocchi^6^, Andrea De Maria^7,8,+^, Anna Casabianca^1,9,+*^

^1^Department of Biomolecular Sciences, Section of Biochemistry and Biotechnology, University of Urbino Carlo Bo, Fano (PU), Italy

^2^Laboratorio Covid, University of Urbino Carlo Bo, Fano (PU), Italy

^3^Unit of Infection Diseases, San Salvatore Hospital, AST Pesaro Urbino, Pesaro (PU), Italy

^4^Department of Biomedical Sciences and Public Health, Marche Polytechnic University (UNIVPM), Ancona (AN), Italy

^5^Department of Biomolecular Sciences, Campus Scientifico Enrico Mattei, University of Urbino Carlo Bo, Urbino (PU), Italy

^6^Department of Biomolecular Sciences, Service of Biostatistics, University of Urbino Carlo Bo, Urbino (PU), Italy

^7^Department of Health Sciences, University of Genova, Genova (GE), Italy

^8^S. C. Malattie Infettive, Azienda Sociosanitaria del Levante n. 5, Regione Liguria, Sarzana (SP), Italy

^9^Department of Pure and Applied Sciences, Section of Chemistry, Environment and Materials, University of Urbino Carlo Bo, Fano (PU), Italy

^*^Corresponding author: [anna.casabianca@uniurb.it](mailto:anna.casabianca@uniurb.it)

^+^these authors contributed equally to this work

**Table S1** List of drugs taken by PWH

| **Full Name** | **Abbreviations** | **Group A**  **(≥3-drugs)** | **Group B**  **(≤2-drugs)** |
| --- | --- | --- | --- |
| Abacavir | ABC | 5 | - |
| Atazanavir | ATV | 1 | 2 |
| Cobicistat | COBI | - | 38 |
| Darunavir | DRV | 8 | 59 |
| Efavirenz | EFV | 6 | - |
| Emtricitabine | FTC | 14 | - |
| Etravirine | ETR | - | 6 |
| Lamivudine | 3TC | 7 | 15 |
| Lopinavir | LPV | 1 | - |
| Nevirapine | NVP | 1 | 2 |
| Raltegravir | RAL | 1 | 10 |
| Ritonavir | RTV | 10 | 23 |
| Saquinavir | SQV | 1 | - |
| Tenofovir disoproxil fumarate | TDF | 14 | - |
| Zidovudine | AZT | 2 | - |

**Table S2** List of antiretroviral therapies by drug classes

| **Group A**  **≥3-drugs (n=21)** | **Group B**  **≤2-drugs (n=66)** |
| --- | --- |
| 2NRTI+NNRTI (10, 47.6%) | PI (38, 57.5%) |
| 2NRTI+PI (10, 47.6%) | PI+INSTI (5, 7.5%) |
| 2NRTI+PI+INSTI (1, 4.6%) | NRTI+PI (15, 22.7%) |
|  | NNRTI+PI (3, 4.5%) |
|  | NNRTI+INSTI (5, 7.5%) |

**
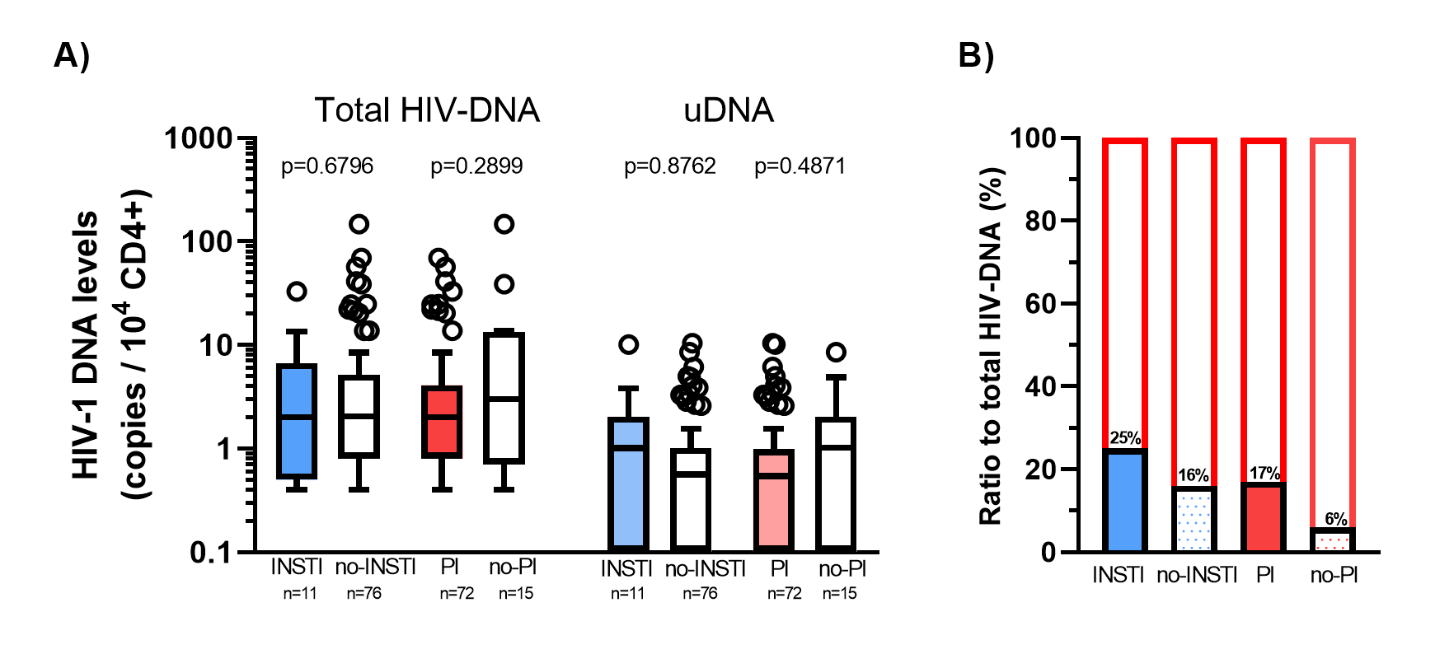
**

**Fig. S1** (A) Comparison of levels of total and unintegrated HIV-DNA in PWH under INSTI-based vs. no-INSTI and PI-based vs. no-PI. (B) Percentages of uDNA among total HIV-DNA in each group. Subtracting uDNA from total HIV-DNA, the remaining fraction consists of integrated HIV-DNA (red line). Mann‒Whitney test

**Table S3** HIV-1 reservoir and immune reconstitution parameters in a subset of 18 PWH on ≤2-drug regimen over time

| **Characteristics** | **T1** | **T2** | ***p* value** ^a^ |
| --- | --- | --- | --- |
| Total HIV-DNA  (copies/10^4 CD4+) | 3.5 [2-5] | 1.1 [0.7-4] | **0.0504** |
| uDNA  (copies/10^4 CD4+) | 0.6 [0.4-1.5] | 0.6 [0-1] | **0.0395** |
| Ratio uDNA to  total HIV-DNA (%) | 25 [16-44] | 25 [0-100] | 0.213 |
| HIV-1 RNA |  |  |  |
| <50 copies/mL of plasma | 15 (83%) | 14 (78%) |  |
| >50 copies/mL of plasma | 3 (17%) | 4 (22%) |  |
| CD4+ count (cells/µL) | 715 [578-1053] | 680 [518-1046] | 0.3225 |
| CD4+ % | 36.7 [26.0-44.6] | 33.5 [23.9-40.4] | 0.7057 |
| CD4/CD8 ratio | 1.07 [0.69-1.56] | 1.03 [0.77-1.26] | 0.3701 |

Results were described by median [IQR] for continuous variables or frequency (%) for categorical variables.

^a^ Wilcoxon matched-pairs signed rank test.

**Table S4** Dynamics of the HIV-DNA, CD4+ T-cell count, CD4+ %, CD4/CD8 ratio and plasma HIV-1 RNA for each of the 18 PWH on ≤2-drug regimen.

| **ID patient** | **Total HIV-DNA (copies/10^4 CD4+)** | | **uDNA (copies/10^4 CD4+)** | | **Ratio uDNA to total HIV DNA (%)** | | **CD4+ T cell count (cells/µL)** | | **CD4+ %** | | **CD4/CD8 ratio** | | **plasma HIV-1 RNA (copies/mL of plasma)** | |
| --- | --- | --- | --- | --- | --- | --- | --- | --- | --- | --- | --- | --- | --- | --- |
|  | **T1** | **T2** | **T1** | **T2** | **T1** | **T2** | **T1** | **T2** | **T1** | **T2** | **T1** | **T2** | **T1** | **T2** |
| Pt_1 | 24.9 | 0.5 | 10.5 | 0.5 | 42% | 100% | 829 | 1037 | 33.0 | 36.9 | ND | 1.15 | <50 | <50 |
| Pt_2 | 1.8 | 0.8 | 0.5 | 0.0 | 25% | 0% | 1388 | 1466 | 44.5 | 47.7 | 1.6 | 1.67 | <50 | <50 |
| Pt_3 | 1.7 | 0.4 | 0.4 | 0.4 | 25% | 100% | 1084 | 1054 | 41.4 | 42.7 | 1.260 | 1.260 | <50 | <50 |
| Pt_4 | 2.0 | 3.3 | 0.5 | 0.4 | 25% | 11% | 636 | 696 | 33.0 | 37.7 | 0.88 | 1.16 | <50 | <50 |
| Pt_5 | 5.1 | 3.8 | 0.8 | 0.0 | 17% | 0% | 578 | 494 | 22.3 | 22.3 | 0.62 | 0.65 | <50 | <50 |
| Pt_6 | 0.4 | 0.4 | 0.0 | 0.0 | 0% | 0% | 1030 | 1261 | 39.6 | 41.3 | 1.52 | 1.74 | <50 | <50 |
| Pt_7 | 3.5 | 0.9 | 0.6 | 0.0 | 17% | 0% | 693 | 541 | 40.5 | 34.1 | 1.32 | 0.99 | <50 | <50 |
| Pt_8 | 3.6 | 0.6 | 0.7 | 0.6 | 20% | 100% | 729 | 761 | 33.7 | 32.9 | 0.92 | 0.86 | <50 | <50 |
| Pt_9 | 2.3 | 2.5 | 0.6 | 0.6 | 25% | 25% | 652 | 680 | 26.0 | 29.0 | 0.75 | ND | <50 | <50 |
| Pt_10 | 4.0 | 1.1 | 1.0 | 1.1 | 25% | 100% | 401 | 368 | 14.0 | 13.8 | 0.23 | 0.21 | <50 | <50 |
| Pt_11 | 5.8 | 0.7 | 0.6 | 0.7 | 10% | 100% | 773 | ND | 48.0 | ND | 1.85 | ND | <50 | <50 |
| Pt_12 | 13.4 | 2.0 | 3.8 | 2.0 | 29% | 100% | 376 | 273 | 26.0 | 20.0 | 0.51 | 0.37 | <50 | <50 |
| Pt_13 | 3.7 | 4.8 | 0.4 | 0.7 | 11% | 14% | 701 | 586 | 45.0 | 37.5 | 1.83 | 1.03 | <50 | <50 |
| **median**  **[IQR]** | **3.6**  **[1.9-5.5]** | **0.9**  **[0.6-2.9]** | **0.6**  **[0.5-0.9]** | **0.5**  **[0.0-0.7]** | **25**  **[14-25]** | **25**  **[0-100]** | **701**  **[607-930]** | **688**  **[506-1050]** | **33.7**  **[26.0-43.0]** | **35.5**  **[24.0-40.4]** | **1.09**  **[0.65-1.58]** | **1.03**  **[0.65-1.26]** | **<50** | **<50** |
| p **value** ^a^ | 0.0156 | | 0.0781 | | 0.0967 | | 0.9697 | | >0.9999 | | 0.7344 | |  | |
|  | | | | | | | | | | | | | | |
| Pt_14 | 0.4 | 1.0 | 0.4 | 1.0 | 100% | 100% | 1241 | 622 | 45.3 | 32.1 | 1.11 | 0.77 | <50 | 92 |
| Pt_15 | 2.4 | 6.7 | 1.2 | 0.0 | 50% | 0% | 325 | 422 | 17.9 | 19.0 | 0.37 | ND | <50 | 644 |
| Pt_16 | 2.1 | 3.9 | 0.3 | 0.3 | 14% | 8% | 1649 | 1215 | 62.5 | 63.6 | 3.94 | 3.98 | 50738 | 429 |
| Pt_17 | 5.8 | 3.9 | 3.9 | 1.0 | 67% | 25% | 578 | 578 | 34.4 | ND | 1.07 | 1.07 | 377 | 685 |
| Pt_18 | 3.5 | 1.1 | 2.6 | 1.1 | 75% | 100% | 1042 | 684 | 39.0 | 31.2 | 0.99 | 0.85 | 60 | <50 |
| **median**  **[IQR]** | **2.4**  **[1.3-4.7]** | **3.9**  **[1.1-5.3]** | **1.2**  **[0.4-3.3]** | **1**  **[0.2-1.1]** | **67**  **[32-88]** | **25**  **[4-100]** | **1042**  **[452-1445]** | **622**  **[500-950]** | **39.0**  **[26.2-53.9]** | **31.7**  **[22.0-55.7]** | **1.07**  **[0.68-2.53]** | **0.96**  **[0.79-3.25]** | **377**  **[60-5x10^4]** | **537**  **[176-675]** |
| *p* **value** ^a^ | >0.9999 | | 0.2500 | | 0.3750 | | 0.2500 | | 0.6250 | | 0.5000 | |  | |
| *p* **value** ^b^ | 0.5160 | 0.0626 | 0.6857 | 0.5140 | 0.0231 | >0.9999 | 0.5805 | 0.7990 | 0.4869 | 0.7703 | 0.9593 | 0.9495 |  | |

^a^ Wilcoxon matched-pairs signed rank test.

^b^ Mann-Whitney test.

**Table S5** Correlation matrix summarizing the correlations between the variables of interest
